# A Comprehensive Report of German Nationwide Inpatient Data on the Post-COVID-19 Syndrome Including Annual Direct Healthcare Costs

**DOI:** 10.3390/v14122600

**Published:** 2022-11-22

**Authors:** Nike Walter, Markus Rupp, Siegmund Lang, Beate Leinberger, Volker Alt, Thilo Hinterberger, Thomas Loew

**Affiliations:** 1 Department for Psychosomatic Medicine, University Hospital Regensburg, 93053 Regensburg, Germany; 2 Department for Trauma Surgery, University Hospital Regensburg, 93053 Regensburg, Germany

**Keywords:** Post-COVID syndrome, healthcare costs, treatment procedures, epidemiology

## Abstract

Background: The aim of this study was to provide a comprehensive overview of German nationwide data including (i) the number of hospitalized Post-COVID Syndrome (PCS) cases including in-hospital mortality rates and intensive care unit treatments, (ii) the main common concomitant diagnoses associated with PCS, (iii) the most frequently performed treatment procedures, and (iv) the annual direct healthcare costs. Methods: The incidence was calculated based on annual ICD-10 diagnosis codes “U09.9!, Post-COVID-19 condition”. Data on concomitant diagnoses, treatment procedures, treatment in an intensive care unit (ICU), in-hospital mortality, the proportion of G-DRGs, and cumulative costs were assessed based on the Institute for the Hospital Remuneration System (InEK) data for 2019. Results: A total of 29,808 PCS inpatients could be identified yielding a prevalence of 5.5%. In total, 1330 (4.5%) in-hospital deaths were recorded, and 5140 (17.2%) patients required ICU treatment. The majority of patients (18.6%) were aged 65–74 years. The most common concomitant diagnoses included pneumonia, critical illness polyneuropathy, dyspnea, chronic fatigue syndrome, and pulmonary embolisms. The most frequently performed procedures were computed tomography of the thorax with contrast medium, whole-body plethysmography, and the monitoring of respiration, heart, and circulation. The cost per case of the G-DRG codes that were analyzed ranged from € 620 ± 377 (E64D, Respiratory insufficiency, one day of occupancy) to € 113,801 ± 27,939 (A06B, Ventilation > 1799 h with complex OR procedure). Total cumulative direct healthcare costs of € 136,608,719 were calculated, resulting in mean costs of € 4583 per case. Conclusion: Post-COVID Syndrome is of major public health importance with substantial financial implications. The present article can support stakeholders in health care systems to foresee future needs and adapt their resource management. Consensus diagnostic criteria and rehabilitation guidelines are highly warranted.

## 1. Introduction

The COVID-19 pandemic has put a tremendous burden on the global healthcare system. While substantial efforts have been made to understand the mechanisms underlying this disease, the long-term sequela remain largely uncertain. Post-COVID syndrome (PCS) was first described in spring 2020 by a patient-led research collaborative surveying prolonged COVID-19 symptoms [1]. According to the National Institute for Health and Care Excellence (NICE), the condition is defined as a continuation of signs and symptoms consistent with COVID-19 for more than 12 weeks, which could not be explained by alternative diagnoses [2]. As diverse terminology, including “long COVID-19” or “post-acute COVID-19”, has been introduced in the literature, the WHO Classification and Terminologies unit responded with the creation of the International Classification of Diseases 10 (ICD-10) and ICD-11 codes for PCS [3]. 

A wide range of prevalence rates has been reported, ranging from 10% to 35% in an outpatient setting [4]; however, a working group from Italy showed that up to 87% of patients experienced the persistence of at least one symptom, especially fatigue and dyspnea, 60 days after recovery from COVID-19 [5]. Other studies revealed that neurocognitive long-COVID symptoms can persist more than one year after COVID-19 onset, reducing patients’ quality of life [6]. PCS has further been associated with a diversity of long-term symptoms such as fatigue, breathlessness, chest pain, cognitive impairment, dyspnea, olfactory and gustatory dysfunction, mental disorders, and insomnia, which can also occur after a relatively mild acute infection [4,5,7,8,9,10]. However, it is important to note that there is no consensus on the definition of PCS in terms of symptoms. 

Moreover, research agendas addressing topics such as treatment, rehabilitation, and chronic care management are highly necessary as guidance for clinical decision-making remains scarce [11,12]. For instance, the so-called REHabilitation COVID-19 Evidence-based Response (REHCOVER) was launched in 2020 with the aim of the rapid dissemination of knowledge on COVID-19 and rehabilitation in the form of systematic reviews [13]. Notably, in their update on the 31 December 2020, they could only identify *n* = 2 out of *n* = 4441 studies reporting on late-onset consequences of COVID-19, and the authors concluded that the lack of high-level evidence studies remains the main limitation [14]. Thus, given that PCS will likely have a substantial public health impact, there is a need to quantify its burden [15,16]. In this stance, epidemiological analyses of large-registry data can be a valuable resource for stakeholders for estimating future demands and developments. 

Therefore, the aim of this study was to provide a comprehensive overview of German nationwide data including (i) the number of hospitalized PCS cases, including in-hospital mortality rates and intensive care unit (ICU) treatments, (ii) the main common concomitant diagnoses associated with PCS, (iii) the most frequently performed treatment procedures, and (vi) the annual direct healthcare costs. 

## 2. Materials and Methods

In this cross-sectional study, data consisting of annual ICD-10 diagnosis codes were retrieved from the Institute for the Hospital Remuneration System (InEK GmbH, Siegburg, Germany). This universal, performance-based, and flat-rate remuneration system was introduced for general hospital services in accordance with Section 17 b of the German Hospital Financing Act (KHG). The basis for this is the G-DRG system (German Diagnosis-Related Groups system), whereby each inpatient case of treatment is remunerated by means of a corresponding DRG lump sum payment. The data was accessed via the InEK Data Browser (https://datenbrowser.inek.org/, accessed on 30 September 2022). The analysis was performed for the year 2021. The number of patients diagnosed with COVID-19 (ICD-10: U07.1!, U07.2!, and U10.9) as well as the number of patients with the secondary diagnosis “U09.9!, Post-COVID-19 condition” were extracted. Incidences were calculated based on Germany’s population provided by the Federal Statistical Office of Germany (Destatis). Here, the number of inhabitants in each of the 16 German federal states was considered by year of birth. Furthermore, the total case numbers of in-hospital deaths, and the number of cases treated in an Intensive Care Unit (ICU) were determined. Additionally, the length of hospital stay, the performed procedures, and the G-DRG codes were analyzed. To estimate the cost for the inpatient treatment of Post-COVID syndrome, the G-DRG Report browser was employed. The distribution of cases according to the Patient Clinical Complexity Level (PCCL) was adopted from the InEK Data Browser. The PCCL value was calculated in a complex procedure from the secondary diagnosis values (complication or comorbidity level values − CCL) and indicates the severity of the complication or comorbidity based on results between 0 (no CC) and 6 (most severe CC). Costs according to the distribution of the applied G-DRG codes, that were used in at least 0.05% of cases, were added up proportionally and calculated as the mean value per case.

## 3. Results

**Figure 1:**
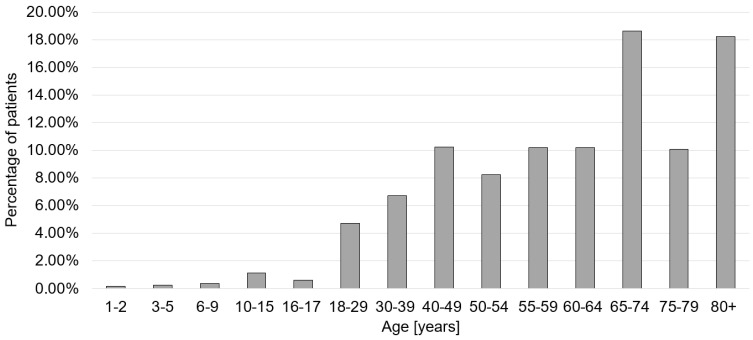
Age distribution of Post-COVID syndrome patients.

**Figure 2:**
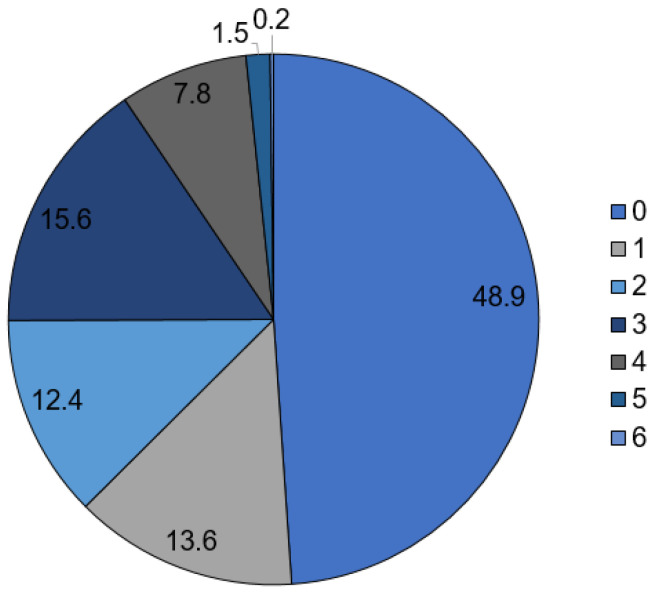
Distribution of the Patient Clinical Complexity Level in association with the Post-COVID syndrome given in percentages.

In 2021, 543,789 patients were hospitalized with a coded diagnosis of COVID-19 (Table 1). Out of these, 123,982 patients (60.6% male) received ICU treatment, whereby the majority of patients were aged 65–74 years (23.5%). The mean ICU stay was 18.9 days. In addition, 69,293 in-house deaths were identified (58.4% male, 53.6% older than 80 years). A total of 29,808 patients could be identified with PCS yielding a prevalence of 5.5%. The majority of patients (18.6%) were aged 65–74 years (Figure 1). In the PCS cohort, 1330 (4.5%) in-hospital deaths were recorded, and 5140 (17.2%) patients required ICU treatment (Table 1). Furthermore, 624 (2.09%) patients received level-1 care, 3055 (10.25%) level-2 care, 2405 (8.07%) level-3 care, 1069 (3.59%) level-4 care, and 5450 (1.51%) level-5 care. The mean length of hospital stay was 11.5 days. In about half of the cases, the clinical complexity was low (Figure 2). 

In total, 865 different main diagnoses in combination with the PCS were identified. The most common ones are listed in Table 2. Further, 1686 different treatment procedures were recorded. Table 3 provides an overview of the thirthy most frequent ones. 

The cost per case of the G-DRG codes that were analysed ranged from € 620 ±377 (E64D, Respiratory insufficiency, one day of occupancy) to € 113,801 ±27,939 (A06B, Ventilation > 1799 h with complex OR procedure). By multiplication of the number of cases with the respective average cost per case according to the used G-DRG code the approximately costs per G-DRG code were calculated. Summed up, in 2019 total costs of € 136,608,719 were produced by the treatment 29,808 inpatients with the PCS. This yields a mean cost of € 4583 per case. G-DRG codes with the highest share of the total cost are shown in Table 4. 

## 4. Discussion

In this cross-sectional study, (i) the number of hospitalized PCS cases including in-hospital mortality rates and intensive care unit (ICU) treatments, (ii) the main common concomitant diagnoses associated with PCS, (iii) the most frequently performed treatment procedures, and (vi) the annual direct healthcare costs were reported for the year 2019. An outstanding characteristic is that the analysis is based on nationwide healthcare insurance data from one of the largest countries of the European Union. 

A total of 29,808 PCS inpatients could be identified yielding a prevalence of 5.5%. In the literature, a wide range of prevalence is reported. In China, prevalence rates up to 37.6% for hospitalized patients were estimated [17], while PCS was detected in half of COVID-19 survivors (*n* = 277) in Spain [18]. In a French cohort of *n* = 120 hospitalized patients, persistent symptoms were reported more than 100 days after admission including fatigue (55%), dyspnoea (42%), and loss of memory (34%) [19]. Furthermore, our results indicated that PCS is not only likely to occur in elderly multimorbid patients, but also in younger patients. Here, approximately 50% of the cohort had a low Patient Clinical Complexity Level, which is in line with other findings [10,20,21,22]. Moreover, it has also been reported that PCS occurs in patients who did not experience severe symptoms during acute infection or even in those who had an asymptomatic course of the disease [9,23]. For non-hospitalized patients, prevalence rates ranging from 7.5% to 41% were identified in a recent meta-analysis [16]. In addition, the presented data showed heterogeneity among concomitant diagnoses, reflecting the cardiovascular risks and neurological impairments, which have been described previously [9]. However, here one has to consider that the symptoms identified in Table 2 may not be solely attributed to PCS. Notably, research is also hindered by the lack of definition criteria with only the vague consensus that this entity refers to persisting symptoms after COVID-19 diagnosis. However, in the acute and post-acute phase of COVID-19, symptoms can be very diverse, also depending on which of the multiple SARS-CoV-2 variants the patient had [14,24]. To this end, clear diagnostic criteria and studies applying the Delphi technique are highly warranted. Therefore, in clinical practice, a comprehensive medical examination is essential. PCS patients should be monitored closely and managed in an interdisciplinary way [25]. The macroeconomic burden of the COVID-19 pandemic has been tremendous, with estimated mean costs of $2990.76 ± 545.98 per case and total direct medical costs of $163.4 billion over the course of the pandemic solely in the U.S. [26,27]. Based on the presented G-DRG code analysis, the cumulative direct healthcare costs of € 136,608,719 were calculated for PCS, resulting in mean costs of € 4583 per case. While multiple studies have been devoted to the socioeconomic analyses of initial infections, data on the treatment costs of PCS remain scarce and thus, this study is one of the first to present PCS-associated healthcare costs. Additionally, one study estimated an amount of an additional $223.60 per month over a six-month post-COVID diagnosis period [28]. It is reasonable to assume that indirect healthcare costs will substantially contribute to the burden of disease in the foreseeable future as long-term systems will prevent patients from returning to work. Moreover, it is well established that ICU treatments in particular are associated with job absenteeism and subsequent unemployment [29] One survey determined that 44% of PCS patients were out of the labor force and that 51% worked fewer hours [30]. Another study conducted in Germany found a long-term sick leave rate of 5.8% among 30 950 patients diagnosed with COVID-19 [31]. It has further been determined that patients in the UK spend a mean of £18.1 on non-prescription drugs and report a quality-adjusted life day loss of 32.9 after six months with PCS [32]. 

This study has several limitations. First of all, this is a purely descriptive report. Secondly, only inpatient data were available, and thus, the estimated numbers are limited in their generalizability to the overall population. In addition, the analysis was based on ICD-10 diagnoses, and further individual patient features for an analysis of the risk factors and the severity of the initial COVID-19 infection could not be derived. The correct coding of diagnosis can be assumed since DRG lump sum payment relies on it, which is strictly controlled by the Medical Service of Health Funds. Moreover, it was assumed that PCS was not diagnosed in an acute phase of COVID-19 as defined in the ICD-10. 

In conclusion, Post-COVID syndrome is of major public health importance with substantial financial implications. The present article can support stakeholders in health systems to foresee future needs and adapt their resource management accordingly. Consensus diagnostic criteria and rehabilitation guidelines are highly necessary. 

## Figures and Tables

**Table 1:** Number of cases diagnosed with COVID-19 and Post-COVID syndrome.

Diagnosis	Total Numbers	Incidence/100,000 Inhabitants	% Male/ Female	% ≤65/>65 Years Old	ICU Treatment	In-House Mortality
**COVID-19**	543,789	801.3	51.1/48.9	43.5/56.5	123,082 (22.6%)	69,293 (12.7%)
**Post-COVID**	29,808	43.9	51.8/48.2	53.1/46.9	5140 (17.2%)	1330 (4.5%)

**Table 2:** Thirty main common primary diagnoses in association with Post-COVID syndrome.

ICD-10 Code	Description	Number of Cases	Percentage
J12.8	Pneumonia due to other viruses	1771	5.94%
G62.80	Critical-illness-Polyneuropathy	1652	5.54%
R06.0	Dyspnea	1614	5.41%
G93.3	Chronic fatigue syndrome	703	2.36%
I26.9	Pulmonary embolism without indication of acute cor pulmonale	681	2.28%
J96.00	Acute respiratory failure, not elsewhere classified: Type I (hypoxic)	679	2.28%
J84.1	Other interstitial lung disease with fibrosis	494	1.66%
R53	Malaise and fatigue	492	1.65%
J96.10	Chronic respiratory insufficiency, not elsewhere classified: Type I (hypoxic)	393	1.32%
R26.8	Other and unspecified disorders of gait and mobility	351	1.18%
I50.14	Left-sided heart failure: with symptoms at rest	339	1.14%
I50.01	Secondary right-sided heart failure	328	1.10%
J18.9	Pneumonia, unspecified	276	0.93%
N39.0	Urinary tract infection	275	0.92%
I50.13	Left-sided heart failure: with discomfort on mild exertion	247	0.83%
J98.4	Other changes in the lungs	239	0.80%
J96.01	Acute respiratory failure, not elsewhere classified: Type II (hypercapnic)	236	0.79%
E86	Volume deficiency	229	0.77%
R07.3	Other chest pain	219	0.73%
J84.8	Other interstitial lung disease not otherwise specified	214	0.72%
J96.11	Chronic respiratory insufficiency, not elsewhere classified: Type II (hypercapnic)	204	0.68%
J18.8	Other pneumonia, causative agent unspecified	200	0.67%
R51	Headache	198	0.66%
J18.1	Lobar pneumonia, unspecified	197	0.66%
R07.4	Chest pain, unspecified	187	0.63%
F48.0	Neurasthenia	176	0.59%
I10.01	Benign essential hypertension	172	0.58%
J80.03	Severe adult respiratory distress syndrome (ARDS).	172	0.58%
G47.31	Obstructive sleep apnoea syndrome	167	0.56%
R42	Immobility	154	0.52%

**Table 3:** Thirty main common procedures performed for Post-COVID syndrome patients.

CPT Code	Description	Number of Cases	Percentage
3–222	Computed tomography of the thorax with contrast medium	5233	17.56%
1–710	Whole-body plethysmography	4990	16.74%
8–930	Monitoring of respiration, heart, and circulation	4968	16.67%
3–990	Computer-aided image data analysis with 3D evaluation	4062	13.63%
3–202	Native computed tomography of the thorax	3431	11.51%
1–711	Determination of CO diffusion capacity	3329	11.17%
3–200	Native computed tomography of the skull	3037	10.19%
1–632.0	Diagnostic esophagogastroduodenoscopy	2379	7.98%
1–620.00	Diagnostic tracheobronchoscopy	2118	7.11%
3–225	Computed tomography of the abdomen with contrast medium	2043	6.85%
8–831.0	Placement of catheter in central venous vessels:	1800	6.04%
8–800.c0	Whole blood transfusion, red blood cell concentrate, and platelet concentrate: Red blood cell concentrate: 1 TE to less than 6 TE	1720	5.77%
8–550.1	Geriatric early rehabilitation complex treatment: at least 14 treatment days and 20 therapy units	1554	5.21%
1–620.01	Diagnostic tracheobronchoscopy with bronchoalveolar lavage	1518	5.09%
1–207.0	Electroencephalography	1413	4.74%
1–715	Guyatt six-minute walk test	1269	4.26%
3–800	Native magnetic resonance imaging of the skull	1261	4.23%
1–204.2	Examination of the cerebrospinal fluid system: lumbar puncture for cerebrospinal fluid sampling	1186	3.98%
1–843	Diagnostic aspiration from the bronchus	1123	3.77%
8–706	Application of a mask for mechanical ventilation	1089	3.65%
1–206	Neurography	1075	3.61%
9–320	Therapy of organic and functional disorders of speech, language, voice, and swallowing	978	3.28%
3–052	Transesophageal echocardiography (TEE)	926	3.11%
8–701	Endotracheal intubation	899	3.02%
3–820	Magnetic resonance imaging of the skull with contrast medium	863	2.90%

**Table 4:** Main common G-DRG codes.

G-DRG Code	Description	Number of Cases	Percentage	Mean Cost Per Case [Euro]	Standard Deviation [Euro]	Overall Costs [Euro]
E69C	Bronchitis and bronchial asthma, one day of occupancy or without extreme severe or severe CC or age < 56 years or respiratory complaints and symptoms or respiratory disorders with cause in the neonatal period, without certain extensive/highly extensive treatment	1923	6.45%	1192	476	2,292,216
E79C	Infections and inflammations of the respiratory organs without complex diagnosis, without extremely serious CC or one day of occupancy, except in the case of para-/tetraplegia, without certain moderately complex treatments	1534	5.15%	1939	842	2,974,426
Z65Z	Complaints, symptoms, other abnormalities, and aftercare	1242	4.17%	1483	752	1,841,886
E64A	Respiratory failure, more than one day of occupancy, with extremely severe CC or pulmonary embolism	814	2.73%	2366	1153	1,925,924
E74Z	Interstitial lung disease	775	2.60%	2070	1027	1,604,250
E42Z	Geriatric early rehabilitative complex treatment for diseases and disorders of the respiratory organs	725	2.43%	4878	1418	3,536,550
F62C	Heart failure and shock without severe CC or without dialysis, without complicated diagnosis, without complicated treatment	636	2.13%	2136	965	1,358,496
E75C	Other diseases of the respiratory organs without extremely severe CC or respiratory complaints and symptoms with a complex diagnosis	523	1.75%	1633	740	854,059
E64C	Respiratory failure, more than one day of occupancy, without extremely severe CC, age > 15 years.	504	1.69%	1846	964	930,384
B43Z	Early rehabilitation for diseases and disorders of the nervous system, more than 27 days	488	1.64%	10,131	3359	4,943,928
E63B	Sleep apnea syndrome or polysomnography or cardiorespiratory polygraphy, up to 2 days of occupancy, age > 17 years	480	1.61%	907	315	435,360
B71D	Diseases of cranial nerves and peripheral nerves without complex diagnosis	449	1.51%	1693	878	760,157
G67C	Esophagitis, gastroenteritis, gastrointestinal hemorrhage, ulcer disease, and various diseases of the digestive organs without specific or other complicating factors	443	1.49%	1284	522	568,812
F71B	Nonsevere cardiac arrhythmia and conduction disorders	439	1.47%	1231	525	540,409
F74Z	Thoracic pain and other unspecified diseases of the circulatory system	421	1.41%	1051	343	442,471
A13H	Ventilation > 95 h with specific OR procedure or complicated constellation.	376	1.26%	7839	3112	2,947,464
B81B	Other diseases of the nervous system without complex diagnosis	345	1.16%	1933	870	666,885
E65C	Chronic obstructive pulmonary disease without extremely severe CC	337	1.13%	1780	798	599,860
E41Z	Early rehabilitation for diseases and disorders of the respiratory organs	316	1.06%	8040	4134	2,540,640
B77Z	Headache	313	1.05%	1467	572	459,171
F75C	Other diseases of the circulatory system without extremely severe CC	303	1.02%	2087	1102	632,361
F49G	Invasive cardiology diagnosis	300	1.01%	1804	648	541,200
B42B	Early rehabilitation for diseases and disorders of the nervous system for up to 27 days without neurological complex treatment of acute stroke	287	0.96%	7913	2311	2,271,031
K62C	Various metabolic diseases	269	0.90%	1581	773	425,289
E40C	Diseases and disorders of the respiratory organs with ventilation > 24 h	256	0.86%	5243	2351	1,342,208
B44B	Geriatric early rehabilitation complex treatment for diseases and disorders of the nervous system with other neurological complex treatment	250	0.84%	4948	1372	1,237,000
L63E	Infections of the urinary organs	236	0.79%	1472	621	347,392
F67C	Hypertension without complicating diagnosis	227	0.76%	1208	473	274,216
A09F	Ventilation > 499 h	225	0.75%	27,472	8194	6,181,200
B44C	Geriatric early rehabilitation complex treatment for diseases and disorders of the nervous system without complex treatment	225	0.75%	4262	972	958,950

## Data Availability

The data that support the findings of this study are available on request from the corresponding author.
